# Ocular Manifestations of Mpox and Other Poxvirus Infections: Clinical Insights and Emerging Therapeutic and Preventive Strategies

**DOI:** 10.3390/vaccines13050546

**Published:** 2025-05-21

**Authors:** Yuan Zong, Yaru Zou, Mingming Yang, Jing Zhang, Zizhen Ye, Jiaxin Deng, Kyoko Ohno-Matsui, Koju Kamoi

**Affiliations:** 1 Department of Ophthalmology, Zhongshan Torch Development Zone People’s Hospital, Zhongshan 528436, China; zongyuan666@gmail.com; 2 Department of Ophthalmology & Visual Science, Graduate School of Medical and Dental Sciences, Institute of Science Tokyo, Tokyo 113-8510, Japan; alicezouyaru519@gmail.com (Y.Z.); yangmm-12@outlook.com (M.Y.); zhangj.c@foxmail.com (J.Z.); yezizhen518@gmail.com (Z.Y.); dengjiaxin.med@gmail.com (J.D.); k.ohno.oph@tmd.ac.jp (K.O.-M.); 3 International Ocular Surface Research Center, Institute of Ophthalmology, and Key Laboratory for Regenerative Medicine, Jinan University Medical School, Guangzhou 510632, China

**Keywords:** mpox, ocular manifestations, poxvirus infections, smallpox vaccine, public health emergency

## Abstract

Poxvirus infections, particularly those caused by the monkeypox virus, have emerged as significant public health threats. Ocular manifestations constitute a severe potential clinical complication associated with these infections, potentially resulting in permanent visual impairment in afflicted patients. This review aimed to examine the clinical spectrum of ocular manifestations associated with mpox and other poxvirus infections and to evaluate current management strategies alongside emerging therapeutic interventions and prevention strategies. A comprehensive literature search was performed across major databases to identify studies reporting ocular involvement in poxviral infections. Ocular involvement in poxviral infections ranges from mild conjunctivitis and eyelid lesions to severe keratitis with potential vision loss. Mpox-related ocular manifestations are more prevalent in unvaccinated and immunocompromised individuals. Although early antiviral intervention and supportive care are critical, clinical outcomes vary considerably across viral clades. Emerging evidence indicates that tecovirimat may reduce lesion severity, although its impact on accelerating recovery remains limited. Moreover, vaccine strategies, particularly the MVA-BN (JYNNEOS) vaccine, appear to decrease ocular complications, despite regional disparities in access and implementation. Ocular complications pose a significant clinical challenge in mpox and related poxviral infections. This review highlights the need for early diagnosis and integrated treatment approaches that combine antiviral therapy, supportive care, and targeted vaccination. Further research is essential to refine treatment protocols and assess the long-term outcomes in diverse patient populations.

## 1. Introduction

Within the family *Poxviridae*, four genera are of significant importance in human pathology: *Orthopoxvirus*, *Parapoxvirus*, *Yatapoxvirus*, and *Molluscipoxvirus* [1]. The *Orthopoxvirus* genus includes the variola virus, which was eradicated in 1980; the vaccinia virus, also part of this genus, plays a crucial role in the production of the smallpox vaccine; and the monkeypox virus (MPXV), a zoonotic virus that causes symptoms similar to those of smallpox, although typically less severe [2,3].

Mpox was previously endemic in parts of Central and Western Africa [4,5]. Nevertheless, it has recently garnered substantial attention because of two unprecedented outbreaks [6,7,8]. The 2022 outbreak primarily spread through Western and European nations, although the Americas also recorded cases. The World Health Organization (WHO) issued a public health emergency of international concern (PHEIC) declaration in July 2022 because of this outbreak [5,6,7]. More recently, a new wave of mpox cases appeared in 2023-2024, primarily affecting African countries. The African Centers for Disease Control and Prevention (CDC) announced mpox as a regional public health emergency, and the WHO issued a second PHEIC declaration in August 2024 [8,9].

Human MPXV infections manifest as a spectrum of ocular complications, necessitating an increased clinical awareness among practitioners, particularly ophthalmologists. The virus may gain ocular access through either autoinoculation or systemic viremia [10,11]. This can cause mild-to-severe eye issues. The common complications include conjunctivitis, blepharitis, and eyelid swelling. Some patients developed keratitis, corneal ulcers, or eyelid lesions. The prevalence of mpox in specific areas leads to eyelid swelling and conjunctivitis, affecting 30% of the unvaccinated infected individuals. The most severe complications from MPXV infection result in corneal scarring and vision loss [7,10]. The 2022 and 2024 mpox outbreaks differ from previous mpox outbreaks that have occurred in endemic regions. The observed differences in MPXV type or transmission patterns could explain these new patterns of infection. The occurrence of ocular complications due to MPXV infection remains less severe in regions where the virus is not endemic [7].

This review focuses specifically on the ocular manifestations of MPXV infection and provides ophthalmologists and other clinicians with critical insights into its clinical presentations, diagnostic approaches, and management strategies. We examine the spectrum of ocular involvement in mpox, compare it with the ocular manifestations of other poxvirus infections, and discuss emerging therapeutic and preventive approaches relevant to eye-care professionals. By concentrating on this understudied aspect of mpox, we aim to address a significant knowledge gap in the literature and provide practical guidance for clinicians in managing the potential ocular complications of this emerging infectious disease.

## 2. MPXV Variants and Transmission

**Figure 1:**
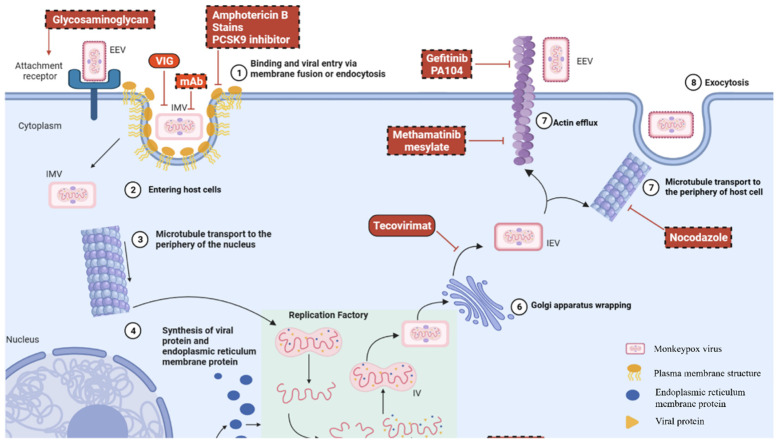
MPXV replication cycle and antiviral targets. This diagram shows the MPXV life cycle from entry to excretion. EEVs and IMVs enter cells via fusion or endocytosis, using glycosaminoglycans as receptors. IMVs are transported to perinuclear replication sites, where their genomes replicate. IMVs are then enveloped by Golgi membranes to form IEVs, which are transported to the cell surface. Key antiviral drugs targeting each stage are indicated. Abbreviations: EEVs, extracellular enveloped virions; IMVs, intracellular mature virions; IEVs, intracellular enveloped virions; IV, immature virion. (Adapted from Ref. [15] under the terms of the Creative Commons Attribution License (CC BY)).

MPXV is transmitted through direct contact with cutaneous lesions, respiratory droplets, bodily fluids, and contaminated fomites, via potential zoonotic and human-to-human transmission routes [12]. During the 2022 global mpox outbreak, contact with skin lesions was identified as the dominant route of transmission [12,13]. The virus subsequently proliferates in the lymphatic system, undergoing two phases of viremia: the first phase is typically asymptomatic, while the second phase results in viral dissemination to peripheral tissues, particularly the skin and mucous membranes [14]. Figure 1 illustrates the life cycle of the MPXV, from cellular entry to egress, highlighting the key antiviral targets at each stage. Both extracellular enveloped virions and intracellular mature virions (IMVs) use glycosaminoglycans as receptors to enter cells via fusion or endocytosis. IMVs are subsequently transported to the perinuclear replication sites, where viral genome replication occurs. Following replication, IMVs are enveloped by the Golgi membrane, forming intracellular enveloped virions, which are transported to the cell surface [15].

MPXV has two main clades, clade I (formerly the Congo Basin clade) and clade II (formerly the West African clade). Clade I includes subclades Ia and Ib, whereas clade II contains subclades IIa and IIb [16,17].

The endemic clade Ia MPXV strain continues to exist in West and Central Africa, where it accounted for nearly all the reported cases before the global clade IIb outbreak began in 2022 [18]. MPXV was first identified in humans in the Democratic Republic of the Congo (DRC) in 1970, and researchers have established its primary zoonotic transmission route from rodents to human hosts [19,20]. The clinical course of this clade produces severe disease manifestations, along with a death rate of up to 10%, primarily affecting children younger than 10 years [21,22].

The newly discovered clade Ib MPXV variant appeared in September 2023 among sex workers in Kamituga DRC [23]. The clade Ib MPXV strain has spread to neighboring countries, including Kenya, Rwanda, Uganda, and Burundi since August 2024. The CDC has recorded over 15,000 mpox cases and 400 deaths in this region in 2024 before the declaration [8,18]. In response to this escalating situation, the WHO issued a second PHEIC declaration on 14 August 2024 [8]. Alarmingly, clade Ib was detected outside Africa in Sweden and Thailand in late August 2024, raising concerns about its potential global spread [24,25,26]. However, the epidemiological patterns of clade Ib remain unclear. It primarily spreads through close contact among heterosexual sex workers. However, young children can also acquire the infection through close contact with infected household members [27].

Clade II MPXV infection is associated with a lower mortality rate but enhanced transmissibility [16,21]. Clade IIa MPXV, endemic to West Africa since its identification in Nigeria in 1971, primarily maintains a zoonotic transmission pattern [19,28]. However, it has occasionally been reported outside Africa [29,30]. While zoonotic transmission remains predominant, human-to-human transmission of clade IIa MPXV has been documented in Nigeria and the United Kingdom [31,32].

The pandemic caused by clade IIb MPXV continued to spread across the world during 2022–2023 [33]. Clade IIb MPXV was first detected in Nigeria in 2017 before scientists identified it as clade IIb lineage A, which showed human-to-human transmission, including sexual transmission [34,35]. A new variant, clade IIb lineage B.1, initiated a worldwide pandemic during 2022–2023 after its first detection in early May 2022 in the United Kingdom, Spain, and other European nations [34,36,37]. The clade showed exceptional human-to-human transmission capabilities, primarily affecting men who have sex with other men through intimate physical contact and sexual networks, resulting in the mpox’s first PHEIC declaration in July 2022 [33]. The combination of public health interventions, behavioral changes, and vaccination campaigns resulted in decreased mpox case numbers, leading to the WHO lifting the PHEIC status in May 2023 [38].

## 3. Systemic Manifestations of Mpox

Understanding the systemic symptoms of mpox is crucial for the differential diagnosis of mpox-related ocular complications, as the latter are often part of the overall disease progression. Mpox typically has an incubation period of up to 21 days, with most patients developing symptoms within 7 to 14 days after exposure [38,39]. The disease generally begins with nonspecific systemic symptoms including fever, headache, myalgia, backache, and fatigue [39,40]. Another distinguishing feature of mpox is lymphadenopathy, which often occurs early during the course [41]. The characteristic rash typically appears 1–5 days after fever onset and progresses from macules to papules, vesicles, pustules, and finally to crusted scabs. The lesions are usually deep-seated, firm, and 2–10 mm in size, typically lasting 2–4 weeks [2,41]. Severe complications have been noted in immunocompromised patients, particularly those with untreated human immunodeficiency virus (HIV)/acquired immunodeficiency syndrome [5,42,43].

The clinical presentations vary significantly according to the viral clade. Clade Ia is associated with more severe disease and higher case fatality rates (up to 25.6%, primarily in unvaccinated children), with rashes often following centrifugal distribution [44]. Clade Ib appears to disproportionately affect children and young adults at high risk of genital lesions [20,22]. Clade IIa is generally milder than clade I, with lower case fatality rates [28,29]. Clade IIb, which is responsible for the 2022 global outbreak, often presents with localized rashes, predominantly in the perianal or genital regions, with initial lesions commonly occurring at sites of oral or anogenital exposure [7,37].

## 4. Ocular Complication in Poxvirus Infections with Focus on Mpox

### 4.1. Ocular Complications in Poxvirus Infections

Prior clinical experience in managing ocular complications of other poxvirus infections can provide valuable insights into understanding and addressing mpox-related ophthalmic manifestations. Poxvirus infections, such as variola virus infection (smallpox), cowpox virus infection, and vaccinia virus infection, have been associated with ocular complications. These infections result in diverse ocular complications ranging from mild to severe conditions that threaten vision. The infection affects different parts of the eye, starting from the eyelids, extending to the conjunctiva and cornea, and reaching the posterior segment [45].

The medical literature shows that variola virus infection frequently results in various eye complications. The periorbital area exhibits distinctive rashes with swelling and excess moisture. The development of pustules on the conjunctiva leads to painful symptoms, alongside photophobia and excessive tearing. Smallpox-related corneal ulceration is one of the most severe ocular complications and can result in perforation, iris prolapse, and hypopyon formation. Posterior segment involvement in smallpox is associated with chorioretinitis, retinitis, and optic neuritis [45,46,47].

Infection with the cowpox virus leads to various eye complications, including swollen eyelids, ulcerations, conjunctivitis, and superficial keratitis. In rare cases, persistent corneal infections can cause corneal melting, requiring corneal transplantation [45,48].

### 4.2. Clinical Features of Mpox-Related Eye Disease

#### 4.2.1. Overview of Ocular Manifestations

Before the first PHEIC declaration for mpox in 2022, the estimated rate of ocular complications from mpox affected 4–5% of patients in Africa [49]. A 2023 meta-analysis conducted during the first PHEIC of mpox [50] showed that the pooled prevalence of ocular manifestations in reported cohorts of patients with mpox was 9% (95% confidence interval (CI): 3–24). The specific ocular complications include photophobia (30.87%, 95% CI: 28.13–33.67), conjunctivitis (13.89%, 95% CI: 6.92–22.67), keratitis/corneal ulceration (3.33%, 95% CI: 1.99–4.95), and visual impairment (7.69%, 95% CI: 5.30–11.03). The presence of ocular involvement in patients is mostly accompanied by systemic symptoms and vesiculopustular lesions [7,11]. However, isolated ocular mpox without systemic symptoms or skin lesions has been reported [50,51]. Unvaccinated and immunocompromised patients develop more serious and frequent ocular complications [7].

#### 4.2.2. Periocular Manifestations

**Figure 2:**
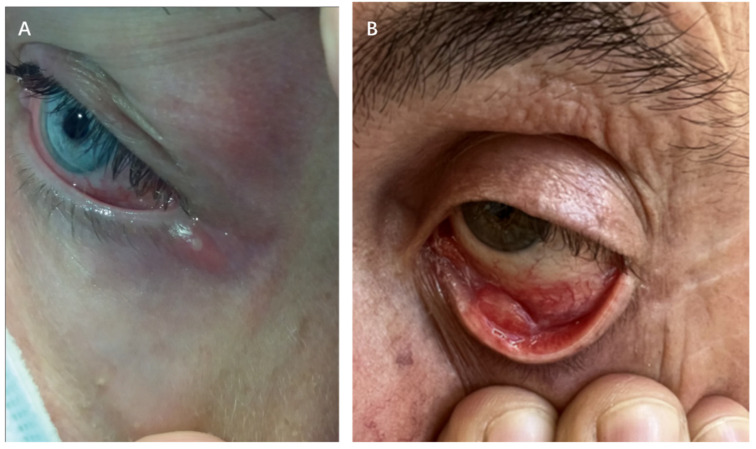
Characteristic conjunctival and eyelid involvement of MPXV infection. (**A**) Multiple conjunctival and periocular vesicular lesions induced by MPXV. (**B**) Ulcerative lesions on the palpebral conjunctiva in a patient with confirmed MPXV infection. ((**A**) Adapted with permission from Ref. [54]. 2022, Benatti et al.; (**B**) Adapted from Ref. [55]. 2022, De Sousa et al. under the terms of the Creative Commons Attribution License (CC BY)).

The periocular area shows vesiculopustular rashes on the eyelids and periorbital skin that match cutaneous lesions found elsewhere on the body (Figure 2A) [10,52]. A retrospective study conducted before the 2022 global mpox outbreak showed that 25% of hospitalized patients with mpox in Nigeria (clade IIa) developed periocular mpox rashes between September 2017 and December 2018 [53].

#### 4.2.3. Anterior Segment Manifestations

##### Conjunctivitis

The predominant ocular manifestation associated with mpox is conjunctivitis, which manifests as diverse clinical presentations, including follicular, serpiginous, ulcerative, nodular, and pseudomembranous forms (Figure 2A,B) [7,11,38]. The exact mechanisms underlying mpox-related conjunctivitis remain unclear. However, two possible mechanisms have been suggested based on current observations: (1) self-transmission from adjacent eyelid margin lesions and (2) development of both eyelid margin and conjunctival lesions as part of the regional infection spread [56]. Some patients present with simultaneous eyelid margin lesions and conjunctivitis, making it difficult to identify the exact cause [57].

The development and evolution of conjunctivitis during MPXV infection depend directly on the immune system of the infected host. MPXV leads to the development of conjunctivitis more easily in people with HIV infection or in children because their immune systems are compromised. A study from the Congo (formerly Zaire) documented the death of an unvaccinated 2.5-year-old child with mpox who developed generalized vesicles and pustules, along with bilateral conjunctivitis and eyelid lesions [58]. The appearance of eye symptoms indicated a worse course of systemic MPXV infection. Patients who developed mpox-related conjunctivitis experienced more systemic symptoms compared with patients without conjunctivitis [7,11].

Furthermore, the diagnosis of bacterial conjunctivitis remains essential even when mpox shows typical systemic features because some suspected mpox-related conjunctivitis cases result in bacterial conjunctivitis [59].

##### Keratitis

**Figure 3:**
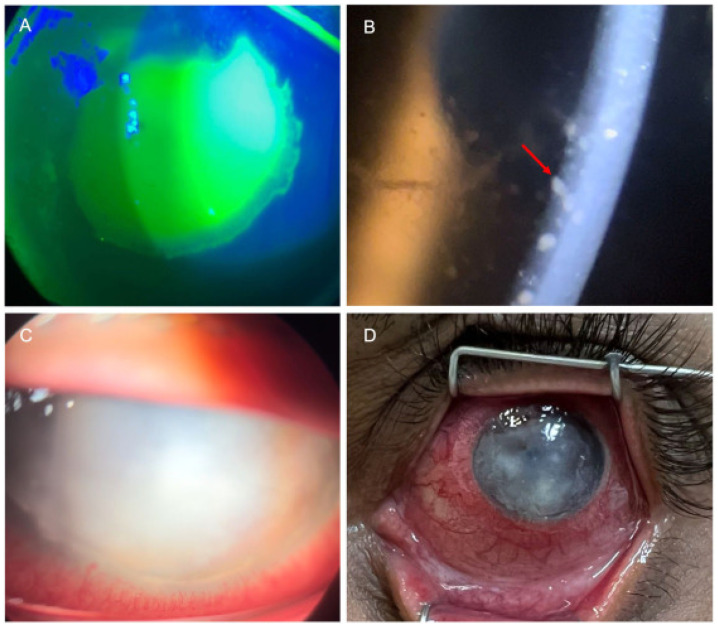
Intraocular manifestations of MPXV infection. (**A**) Epithelial keratitis with fluorescein staining highlighting corneal involvement. (**B**) Anterior uveitis with granulomatous keratic precipitates (arrow). (**C**) Keratitis with significant corneal edema and hypopyon. (**D**) Diffuse ocular hyperemia with keratitis and corneal opacities. (Adapted with permission from Ref. [64], 2024, Finamor et al.).

Keratitis, particularly ulcerative keratitis, can lead to corneal scarring and significant visual impairment (Figure 3A,C,D) [60]. In the DRC monitoring from 1980 to 1988 [61], 4.3% (12 of 282) of patients with clade Ia mpox developed keratitis or corneal ulceration. Domínguez García et al. [62] documented an instance of persistent and severe mpox-associated keratitis in Spain in 2024, specifically ulcerative keratitis in an immunocompetent individual, which persisted despite the administration of both systemic and topical interventions, including antiviral therapies and amniotic membrane transplantation. Arcuate serpiginous centripetal epithelial keratitis appears to represent a distinctive form of keratitis specifically associated with MPXV infection [62,63]. Additionally, among other anterior segment manifestations, it has been reported that severe and persistent ulcerative keratitis is accompanied by uveitis [63].

##### Scleritis

Medical reports document mpox-related scleritis occurring alongside conjunctivitis, keratitis, or uveitis [7,38]. Nguyen et al. reported the case of a 53-year-old HIV-negative man with chronic lymphocytic leukemia and persistent lymphopenia who developed isolated ocular mpox without skin lesions or systemic prodromal symptoms in the USA in 2022 [51]. The patient developed severe scleritis, keratitis, and uveitis in the right eye. Despite negative results from extensive testing for infectious etiologies, metagenomic RNA sequencing identified MPXV RNA in the aqueous humor, and polymerase chain reaction (PCR) confirmed the presence of the virus in the cornea and sclera.

#### 4.2.4. Intraocular Manifestations

##### Uveitis

Uveitis linked to MPXV infection presents predominantly as anterior uveitis (Figure 3B) [50,64]. Carvalho et al. reported a 28-year-old Brazilian male homosexual with mpox who presented with anterior uveitis and conjunctival vesicles [65]. The patient experienced a painless reduction in visual acuity in the right eye, with a visual acuity of 20/30. Examination revealed small keratic precipitates, +1 anterior chamber cells, and discrete cells in the anterior vitreous region. The patient was treated with topical eye corticosteroids and achieved visual acuity of 20/20 in both eyes within 10 days of treatment. The extended use of cidofovir for mpox treatment leads to drug-induced uveitis and ocular hypotony, requiring clinicians to carefully consider cidofovir prescription for mpox treatment [50].

#### 4.2.5. Severe Complications

In extreme cases, immunocompromised patients infected with both MPXV and acquired immunodeficiency syndrome experience devastating panfacial gangrene with ocular rupture, leading to fatal outcomes due to systemic complications [66].

### 4.3. Clade-Specific Ocular Manifestations

MPXV clades (Ia, Ib, IIa, and IIb) exhibit notable differences in ocular manifestations, particularly in the prevalence and severity of conjunctivitis and keratitis. Table 1 presents the ocular manifestations observed across distinct clades in the mpox cohort. The ocular involvement rate is higher in infections caused by clade I. Currently, available statistical data on clades Ib and IIa ocular involvement remain insufficient.

The global outbreak of clade IIb infections demonstrates significantly reduced eye involvement compared with clade Ib infections, particularly in developed countries [13,68,69,70,71]. However, cohorts from developing countries still report relatively high rates of ocular involvement with clade II infections [72,73].

Two notable examples of high ocular involvement rates in clade II outbreaks with distinct characteristics have been reported in Mexico and Nigeria. In a Nigerian cohort [73], only 5% of reported mpox patients self-identified as men who have sex with men, with an overall male-to-female ratio of 114:46. Moreover, 6.3% of patients developed keratitis. This demographic profile differed significantly from the first PHEIC induced by clade IIb MPXV in 2022–2023, which predominantly affected men who have sex with men and featured a lower incidence of keratitis. This may be attributed to the fact that the outbreak in Nigeria is linked to clade IIb lineage A rather than the clade IIb lineage B.1 observed in other regions [18,34]. In another study involving a Mexican cohort [72], most patients with mpox (9/11) and ocular involvement were HIV-positive. These findings suggest that factors, such as HIV co-infection and regional variations in transmission patterns, may influence the prevalence of ocular involvement in clade II MPXV infections.

## 5. Advances and Challenges in the Diagnosis of Mpox-Related Ocular Complications

Accurate diagnosis of mpox is the first line of defense against epidemic outbreaks and severe complications, relying primarily on three elements: physical examination, contact history, and laboratory testing [74]. The systemic manifestations and transmission routes of mpox have been previously described. However, these clinical features are nonspecific and may overlap with symptoms of other infectious diseases, necessitating laboratory testing for confirmation. Nucleic acid amplification tests, particularly real-time PCR, are primarily used. Owing to its high sensitivity and specificity, real-time PCR is considered the gold standard for diagnosing MPXV infection [74,75]. PCR testing is conducted on samples collected from skin lesions, blood, or mucosal sites [75,76]. Serological tests that detect IgM and IgG antibodies can also aid in diagnosis, especially when nucleic acid testing is not feasible. However, these tests may be limited by cross-reactivity and interference from prior vaccination or exposure to other orthopoxviruses [75]. Virus isolation and whole-genome sequencing can further confirm the diagnosis and provide epidemiological information, but they are constrained by technical complexity and biosafety requirements [75,77].

The diagnosis of ocular mpox faces multiple obstacles, and the manifestations of conjunctivitis, blepharitis, and subconjunctival nodules are easily confused with other common ocular infections, such as herpes simplex viral conjunctivitis, necessitating laboratory testing for differentiation [78]. It is important to note that systemic samples, such as blood, are more prone to missed diagnoses due to low viral loads (Ct values > 36); therefore, PCR technology for diagnosing ocular tissue samples (including conjunctival swabs and meibomian gland secretions) remains the gold standard for diagnosing mpox-related ocular complications and should be prioritized for mpox cases presenting with ocular symptoms [7,74]. For patients presenting with conjunctival hyperemia, follicular reactions, or vesicular signs, conjunctival samples should be prioritized for PCR testing. It is noteworthy that the detection of viral DNA in ocular specimens not only confirms infection but also suggests a potential risk of transmission. Meduri et al. have reported a case of unilateral conjunctivitis with MPXV-positive conjunctival swabs in a 39-year-old man who developed red eyes and itchiness five days after a positive mpox PCR swab from cutaneous lesions on his chin and lip. A slit-lamp examination revealed a conjunctival follicular reaction and small white vesicles in the nasal bulbar conjunctiva. Two separate conjunctival PCR swabs were positive for mpox, indicating similar viral loads on the conjunctiva and ocular secretions as the cutaneous lesions [79]. This suggests a potential risk of MPXV transmission through ocular contact, necessitating strict adherence to biosafety protocols during sampling and ophthalmological examinations.

## 6. Current and Emerging Therapeutic Strategies for Ocular MPXV Infection

### 6.1. Management of Systemic Mpox

The progression of mpox is generally self-limiting, and its management primarily involves supportive care [56]. Adequate nutrition, supplementation, and hydration are essential for preventing complications. Short-term systemic corticosteroid therapy (prednisone 30–40 mg/day for three days, followed by 15–20 mg/day for three days) has demonstrated efficacy in controlling mucositis, inflammation, and pain without exacerbating the skin lesions [68]. For severe cases and immunocompromised patients, smallpox-specific medications, including brincidofovir, cidofovir, and vaccinia immune globulin (VIG), are required [79].

Antiviral therapy for mpox remains under investigation, with no established standardized treatment protocol. Figure 1 illustrates the key antiviral agents that target each stage of the MPXV replication cycle. Tecovirimat is the most extensively studied antiviral agent that inhibits viral particle formation by targeting the orthopoxvirus-specific p37 protein [80,81]. Regulatory approval has been obtained from multiple jurisdictions. The United States and Canada have approved tecovirimat for smallpox treatment, while the European Union and the United Kingdom have authorized its emergency use for smallpox, mpox, cowpox, and complications from smallpox vaccination [82]. In January 2025, based on data from 15 clinical trials involving over 800 participants, Japan’s Pharmaceuticals and Medical Devices Agency approved tecovirimat for adults and pediatric patients weighing more than 13 kg.

Despite the promising results from early observational studies, the PALM007 and STOMP randomized controlled trials yielded disappointing outcomes, demonstrating that tecovirimat failed to accelerate lesion healing or reduce viral load [83]. Nevertheless, tecovirimat maintains a favorable safety profile, with good tolerability. The most common adverse effects include fatigue, headache, and nausea; severe adverse events are rare [83].

### 6.2. Ocular Treatment for Mpox

Medical professionals lack standardized treatment approaches for the management of mpox-related ocular manifestations. The predominant management strategy consists of providing supportive care and controlling symptoms [7]. Several therapeutic interventions have been examined for the clinical management of mpox-related ocular manifestations.

Emerging evidence has demonstrated that tecovirimat exhibits therapeutic efficacy against the ocular manifestations of cowpox virus infection while also demonstrating potential clinical utility in the management of mpox-associated ophthalmic complications [7,45]. During the first mpox PHEIC, in Spain [68], researchers treated five patients with severe mpox eye symptoms, including corneal ulcers, stromal swelling, and conjunctivitis, using tecovirimat. The patients experienced significant symptom relief within one week of treatment initiation, with a median recovery time of 29 days (range 25–39 days). No adverse effects were observed during the study period.

The medical community remains divided regarding the use of corticosteroid eye drops. The preventive use of corticosteroid eye drops has been successful in stopping corneal infections in patients with smallpox; however, researchers remain concerned about their ability to slow viral elimination and worsen infection [84]. Research on patients with cowpox has demonstrated that corneal damage occurred after viral elimination, possibly because of corticosteroid medication [48]. A study on *Vaccinia virus* keratitis in animals has demonstrated disease recurrence when researchers stopped using trifluridine and prednisolone [85]. In clinical practice, administering topical corticosteroids to patients before antiviral treatment may exacerbate the severity and persistence of their clinical conditions. Domínguez García et al. [62] have reported a case of mpox keratitis in a 54-year-old immunocompetent male patient who developed conjunctivitis in his left eye 15 days after being diagnosed with mpox mucocutaneous lesions. The patient subsequently received treatment with 0.1% dexamethasone and 0.3% tobramycin eye drops for two weeks. However, two weeks after discontinuing the medication, the patient developed peripheral ulcerative keratitis and epithelial defects, with corneal cultures and PCR confirming mpox keratitis. Despite receiving two courses of trifluridine, two courses of oral tecovirimat, and intravenous cidofovir as antiviral treatments, keratitis persisted for over six months, and the corneal PCR results remained positive. Ultimately, the patient underwent amniotic membrane transplantation.

The treatment protocol includes the administration of 5% povidone-iodine for ocular irrigation, which benefits from its antimicrobial spectrum [86]. The CDC recommends the use of trifluridine eye drops as a potential treatment for MPXV ocular infections. However, trifluridine is associated with adverse ocular effects, including eye irritation, dry-eye syndrome, and corneal damage [38]. Additionally, previous evidence on ocular vaccinia infections suggests that the combined use of trifluridine and topical corticosteroids should be avoided [38,84].

### 6.3. Vaccine Application for Mpox

The smallpox vaccines have been approved for prophylactic use against MPXV infection owing to their demonstrated cross-protective efficacy against *orthopoxviruses* [41,85]. The authorized vaccines for mpox prevention include MVA-BN (JYNNEOS/Imvamune/Imvanex), LC16-KMB, and ACAM2000 [85,87]. The highly attenuated MVA-BN vaccine shows strong mpox prevention capabilities by providing 76% protection after a single dose, which increases to 82% after two doses [87]. The vaccines JYNNEOS (MVA-BN) and ACAM2000^®^ are authorized for mpox prevention and can be used for both pre-exposure prophylaxis and post-exposure prophylaxis. For post-exposure prophylaxis, the vaccine should ideally be administered within four days of exposure to prevent disease onset [85]. Vaccines are considered safe for most people but require specific precautions for pregnant individuals and those with compromised immune systems. ACAM2000 should not be administered to patients with severe immunodeficiency or pregnant individuals because it may cause harmful side effects [88]. JYNNEOS is a safer option for these populations because it lacks replicating properties [89]. Breakthrough infections following vaccination tend to result in less severe clinical symptoms. Historical data from smallpox vaccination records show that approximately 1 in 40,000 vaccine recipients may develop ocular complications. Autoinoculation incidents occur frequently in preschool children and lead to most complications stemming from smallpox vaccination [47,90,91].

Despite these encouraging findings, ongoing research remains imperative to refine vaccination strategies, particularly for at-risk groups, and to evaluate the long-term efficacy of these vaccines against mpox. Regarding ocular complications associated with the vaccination, most smallpox vaccine-related ocular manifestations present as vesicular or pustular lesions involving the eyelids and conjunctiva, with rare reported cases demonstrating corneal involvement [91]. In immunocompetent individuals, these conditions are predominantly self-limiting; however, severe cases may require therapeutic intervention. The recommended treatment modalities include topical antiviral agents (trifluridine or vidarabine), VIG, topical antibiotics, and topical corticosteroids [91]. However, it is imperative to note that the administration of VIG is contraindicated in cases of isolated keratitis induced by smallpox vaccination, because it may exacerbate corneal disease and increase the risk of corneal scarring [85,92]. For vaccine-induced corneal lesions, combination therapy employing antiviral medications and corticosteroids has demonstrated efficacy in mitigating inflammatory responses and minimizing scar formation [91].

## 7. Future Directions in the Prevention and Management of Mpox Ocular Manifestations

Future research directions for the management of mpox-related ocular manifestations are expected to significantly benefit from advances in biomedical technology and interdisciplinary approaches. Advances in molecular techniques, such as the identification of ocular-specific biomarkers and microRNA profiles, may enable earlier detection and more precise intervention for ocular involvement [93]. Nanotechnology offers promising avenues, with nanoparticles, such as silver and gold, demonstrating antiviral properties, as well as the potential to enhance drug delivery and vaccine stability. Silver nanoparticles are currently under investigation for their ability to reduce the infectivity of MPXV [93], and their efficacy in inhibiting ocular infection-related microorganisms has been extensively validated, suggesting their potential utility in combating MPXV intraocular infections [94]. Incorporating a “One Health” approach will remain essential, as controlling zoonotic transmission and early detection can prevent the spread of ocular and systemic manifestations of mpox [95]. The ocular tropism of the newly identified MPXV clade Ib variant remains unverified. However, given the established ocular pathogenicity of clade Ia and the relatively underdeveloped healthcare infrastructure in endemic regions, this emerging lineage warrants vigilant surveillance regarding its potential ocular manifestations. Multidisciplinary collaborations integrating molecular biology, nanotechnology, ophthalmology, and public health will be the key to advancing effective prevention and treatment strategies for mpox-related eye complications in the future.

## 8. Conclusions

The combination of viral evolution and host immune responses creates a vital medical situation that affects the clinical management of MPXV-associated eye diseases. The ocular complications from clade I strains that circulate endemically result in severe eye infections, which primarily affect children; however, clade IIb strains that spread worldwide produce milder eye symptoms yet have become more severe in resource-constrained areas. Early antiviral treatment is essential for managing MPXV ocular tropism, which develops through self-inoculation, hematogenous spread, and conjunctival receptor binding. However, it shows limited clinical success despite its in vitro effectiveness. The use of corticosteroids remains debatable because animal studies have shown that viral persistence occurs after vaccination when keratitis returns. The MVA-BN vaccination strategy decreases eye complications yet fails to reach all areas where the virus is endemic. The rapid spread of clade Ib throughout Africa remains a concern because its effects on the eyes remain unknown. Additional research should investigate how MPXV spreads through the eyes and identify biomarkers specific to different clades because it represents an evolving animal-to-human disease-transfer problem.

## Figures and Tables

**Table 1:** Comparison of ocular manifestations across distinct clades of mpox clades.

Clade	Region/Study	Year	Ocular Involvement Rate	Population Characteristics
Ia	Democratic Republic of the Congo (Hughes et al., 2014) [67]	2010–2013	23.1% conjunctivitis	General population
Ia	Democratic Republic of the Congo (Jezek et al., 1987) [61]	1980–1985	4.3% (12/282) keratitis or corneal ulceration	General population
IIb	Spain (Català et al., 2022) [13]	2022	1.1% (2/185) Periorbital lesions	Predominantly MSM
IIb	Spain (Pazos et al., 2023) [68]	2023	1.0% (9/880) Conjunctivitis, 8.0% (7/880) blepharitis, 0.6% (5/880) eyelid lesions	Predominantly MSM
IIb	UK (Patel et al., 2022) [69]	2022	1.0% (2/197) conjunctivitis	Predominantly MSM
IIb	France (Mailhe et al., 2023) [70]	2022	0.8% (2/264) ocular involvement: 1 with eyelid lesions; 1 with keratitis, conjunctivitis and blepharitis	Predominantly MSM
IIb	France (Doan et al., 2023) [71]	2023	0.3% (2/588) keratitis	Predominantly MSM
IIb	Mexico (Rodriguez-Badillo et al., 2024) [72]	2022	6% (6/100) conjunctivitis, 6% (6/100) eyelid lesions, 1% (1/100) episcleritis, 1% (1/100) keratitis	81.8% of ocular cases were HIV-positive
IIb (A lineage)	Nigeria (Ogoina et al., 2023) [73]	2022–2023	6.3% (10/160) keratitis	5% MSM, M:F ratio 114:46

Abbreviation: MSM: men who have sex with men. HIV: human immunodeficiency virus.

## References

[1] Hata D.J., Powell E.A., Starolis M.W., Realegeno S.E. (2024). What the pox? Review of poxviruses affecting humans. J. Clin. Virol..

[2] Aljabali A.A.A., Obeid M.A., Nusair M.B., Hmedat A., Tambuwala M.M. (2022). Monkeypox virus: An emerging epidemic. Microb. Pathog..

[3] Jacobs B.L., Langland J.O., Kibler K.V., Denzler K.L., White S.D., Holechek S.A., Wong S., Huynh T., Baskin C.R. (2009). Vaccinia virus vaccines: Past, present and future. Antivir. Res..

[4] León-Figueroa D.A., Bonilla-Aldana D.K., Pachar M., Romaní L., Saldaña-Cumpa H.M., Anchay-Zuloeta C., Diaz-Torres M., Franco-Paredes C., Suárez J.A., Ramirez J.D. (2022). The never-ending global emergence of viral zoonoses after COVID-19? The rising concern of monkeypox in Europe, North America and beyond. Travel Med. Infect. Dis..

[5] Hatami H., Jamshidi P., Arbabi M., Safavi-Naini S.A.A., Farokh P., Izadi-Jorshari G., Mohammadzadeh B., Nasiri M.J., Zandi M., Nayebzade A. (2023). Demographic, Epidemiologic, and Clinical Characteristics of Human Monkeypox Disease Pre- and Post-2022 Outbreaks: A Systematic Review and Meta-Analysis. Biomedicines.

[6] Nuzzo J.B., Borio L.L., Gostin L.O. (2022). The WHO Declaration of Monkeypox as a Global Public Health Emergency. JAMA.

[7] Zong Y., Kamoi K., Zhang J., Yang M., Ohno-Matsui K. (2023). Mpox (Monkeypox) and the Eye: Ocular Manifestation, Diagnosis, Treatment and Vaccination. Viruses.

[8] Taylor L. (2024). WHO and African CDC declare mpox a public health emergency. BMJ.

[9] Azizi A., Rose K., Kamuyu G., Ogbeni D., Bernasconi V. (2025). Preparedness and priority research to tackle the mpox outbreak response. Nat. Med..

[10] Abdelaal A., Serhan H.A., Mahmoud M.A., Rodriguez-Morales A.J., Sah R. (2023). Ophthalmic manifestations of monkeypox virus. Eye.

[11] Yi-Ting L., Chien-Hsien H., Hwa-Hsin F., Cheng-Kuo C., Pai-Huei P. (2024). Monkeypox-related ophthalmic disease. Taiwan J. Ophthalmol..

[12] Alcamí A. (2023). Pathogenesis of the circulating mpox virus and its adaptation to humans. Proc. Natl. Acad. Sci. USA.

[13] Català A., Clavo-Escribano P., Riera-Monroig J., Martín-Ezquerra G., Fernandez-Gonzalez P., Revelles-Peñas L., Simon-Gozalbo A., Rodríguez-Cuadrado F.J., Castells V.G., de la Torre Gomar F.J. (2022). Monkeypox outbreak in Spain: Clinical and epidemiological findings in a prospective cross-sectional study of 185 cases*. Br. J. Dermatol..

[14] Bhardwaj P., Sarkar S., Mishra R. (2024). Mpox and related poxviruses: A literature review of evolution, pathophysiology, and clinical manifestations. Asian Pac. J. Trop. Biomed..

[15] Lu J., Xing H., Wang C., Tang M., Wu C., Ye F., Yin L., Yang Y., Tan W., Shen L. (2023). Mpox (formerly monkeypox): Pathogenesis, prevention and treatment. Signal Transduct. Target. Ther..

[16] Happi C., Adetifa I., Mbala P., Njouom R., Nakoune E., Happi A., Ndodo N., Ayansola O., Mboowa G., Bedford T. (2022). Urgent need for a non-discriminatory and non-stigmatizing nomenclature for monkeypox virus. PLoS Biol..

[17] Olawade D.B., Wada O.Z., Fidelis S.C., Oluwole O.S., Alisi C.S., Orimabuyaku N.F., Clement David-Olawade A. (2024). Strengthening Africa’s response to Mpox (monkeypox): Insights from historical outbreaks and the present global spread. Sci. One Health.

[18] Van Dijck C., Hoff N.A., Mbala-Kingebeni P., Low N., Cevik M., Rimoin A.W., Kindrachuk J., Liesenborghs L. (2023). Emergence of mpox in the post-smallpox era-a narrative review on mpox epidemiology. Clin. Microbiol. Infect..

[19] Cho C.T., Wenner H.A. (1973). Monkeypox virus. Bacteriol. Rev..

[20] Srivastava S., Laxmi, Sharma K., Sridhar S.B., Talath S., Shareef J., Mehta R., Satapathy P., Sah R. (2024). Clade Ib: A new emerging threat in the Mpox outbreak. Front. Pharmacol..

[21] Gigante C.M., Korber B., Seabolt M.H., Wilkins K., Davidson W., Rao A.K., Zhao H., Smith T.G., Hughes C.M., Minhaj F. (2022). Multiple lineages of monkeypox virus detected in the United States, 2021–2022. Science.

[22] Vakaniaki E.H., Kacita C., Kinganda-Lusamaki E., O’Toole Á., Wawina-Bokalanga T., Mukadi-Bamuleka D., Amuri-Aziza A., Malyamungu-Bubala N., Mweshi-Kumbana F., Mutimbwa-Mambo L. (2024). Sustained human outbreak of a new MPXV clade I lineage in eastern Democratic Republic of the Congo. Nat. Med..

[23] Masirika L.M., Udahemuka J.C., Schuele L., Ndishimye P., Otani S., Mbiribindi J.B., Marekani J.M., Mambo L.M., Bubala N.M., Boter M. (2024). Ongoing mpox outbreak in Kamituga, South Kivu province, associated with monkeypox virus of a novel Clade I sub-lineage, Democratic Republic of the Congo, 2024. Eurosurveillance.

[24] Branda F., Ceccarelli G., Ciccozzi M., Scarpa F. (2024). First cases of mpox Clade I outside of Africa: Genetic insights on its evolution. Infect. Dis..

[25] Lee S.S., Traore T., Zumla A. (2024). The WHO mpox public health emergency of international concern declaration: Need for reprioritisation of global public health responses to combat the MPXV Clade I epidemic. Int. J. Infect. Dis..

[26] Treutiger C.-J., Filén F., Rehn M., Aarum J., Jacks A., Gisslén M., Sturegård E., Karlberg M.L., Karlsson Lindsjö O., Sondén K. (2024). First case of mpox with monkeypox virus clade Ib outside Africa in a r eturning traveller, Sweden, August 2024: Public health measures. Eurosurveillance.

[27] Petersen E., Hvid U., Tomori O., Pedersen A.G., Wallinga J., Pebody R., Cenciarelli O., Aavitsland P., Van Laeken D., Andreasen V. (2024). Possible scenarios for the spread of mpox outside the endemic focus in Africa. Int. J. Infect. Dis..

[28] Faye O., Pratt C.B., Faye M., Fall G., Chitty J.A., Diagne M.M., Wiley M.R., Yinka-Ogunleye A.F., Aruna S., Etebu E.N. (2018). Genomic characterisation of human monkeypox virus in Nigeria. Lancet Infect. Dis..

[29] Alakunle E., Moens U., Nchinda G., Okeke M.I. (2020). Monkeypox Virus in Nigeria: Infection Biology, Epidemiology, and Evolution. Viruses.

[30] Sejvar J.J., Chowdary Y., Schomogyi M., Stevens J., Patel J., Karem K., Fischer M., Kuehnert M.J., Zaki S.R., Paddock C.D. (2004). Human monkeypox infection: A family cluster in the midwestern United States. J. Infect. Dis..

[31] Vaughan A., Aarons E., Astbury J., Brooks T., Chand M., Flegg P., Hardman A., Harper N., Jarvis R., Mawdsley S. (2020). Human-to-Human Transmission of Monkeypox Virus, United Kingdom, October 2018. Emerg. Infect. Dis..

[32] Yinka-Ogunleye A., Aruna O., Dalhat M., Ogoina D., McCollum A., Disu Y., Mamadu I., Akinpelu A., Ahmad A., Burga J. (2019). Outbreak of human monkeypox in Nigeria in 2017–18: A clinical and epidemiological report. Lancet Infect. Dis..

[33] Burki T. (2022). What does it mean to declare monkeypox a PHEIC?. Lancet Infect. Dis..

[34] Beiras C.G., Malembi E., Escrig-Sarreta R., Ahuka S., Mbala P., Mavoko H.M., Subissi L., Abecasis A.B., Marks M., Mitja O. (2025). Concurrent outbreaks of mpox in Africa-an update. Lancet.

[35] Ogoina D., Izibewule J.H., Ogunleye A., Ederiane E., Anebonam U., Neni A., Oyeyemi A., Etebu E.N., Ihekweazu C. (2019). The 2017 human monkeypox outbreak in Nigeria-Report of outbreak experience and response in the Niger Delta University Teaching Hospital, Bayelsa State, Nigeria. PLoS ONE.

[36] Kraemer M.U.G., Tegally H., Pigott D.M., Dasgupta A., Sheldon J., Wilkinson E., Schultheiss M., Han A., Oglia M., Marks S. (2022). Tracking the 2022 monkeypox outbreak with epidemiological data in real-time. Lancet Infect. Dis..

[37] Orviz E., Negredo A., Ayerdi O., Vázquez A., Muñoz-Gomez A., Monzón S., Clavo P., Zaballos A., Vera M., Sánchez P. (2022). Monkeypox outbreak in Madrid (Spain): Clinical and virological aspects. J. Infect..

[38] Begley J., Kaftan T., Song H., Fashina T., Hartley C.D., Nguyen N., Crozier I., Mwanza J.C., Yeh S. (2024). Ocular Complications of Mpox: Evolving Understanding and Future Directions. Int. Ophthalmol. Clin..

[39] Kaler J., Hussain A., Flores G., Kheiri S., Desrosiers D. (2022). Monkeypox: A Comprehensive Review of Transmission, Pathogenesis, and Manifestation. Cureus.

[40] Chen N., Li G., Liszewski M.K., Atkinson J.P., Jahrling P.B., Feng Z., Schriewer J., Buck C., Wang C., Lefkowitz E.J. (2005). Virulence differences between monkeypox virus isolates from West Africa and the Congo basin. Virology.

[41] Titanji B.K., Hazra A., Zucker J. (2024). Mpox Clinical Presentation, Diagnostic Approaches, and Treatment Strategies: A Review. JAMA.

[42] Khamees A.A., Awadi S., Al-Shami K., Alkhoun H.A., Al-Eitan S.F., Alsheikh A.M., Saeed A., Al-Zoubi R.M., Zoubi M.S.A. (2023). Human monkeypox virus in the shadow of the COVID-19 pandemic. J. Infect. Public Health.

[43] McCollum A.M., Shelus V., Hill A., Traore T., Onoja B., Nakazawa Y., Doty J.B., Yinka-Ogunleye A., Petersen B.W., Hutson C.L. (2023). Epidemiology of Human Mpox—Worldwide, 2018–2021. MMWR Morb. Mortal. Wkly. Rep..

[44] Ogoina D., Damon I., Nakoune E. (2023). Clinical review of human mpox. Clin. Microbiol. Infect..

[45] Fashina T., Huang Y., Thomas J., Conrady C.D., Yeh S. (2022). Ophthalmic Features and Implications of Poxviruses: Lessons from Clinical and Basic Research. Microorganisms.

[46] Baker A.R. (1903). Eye Complications of Smallpox. Some Observations During the Recent Epidemic in Cleveland. J. Am. Med. Assoc..

[47] Semba R.D. (2003). The Ocular Complications of Smallpox and Smallpox Immunization. Arch. Ophthalmol..

[48] Graef S., Kurth A., Auw-Haedrich C., Plange N., Kern W.V., Nitsche A., Reinhard T. (2013). Clinicopathological Findings in Persistent Corneal Cowpox Infection. JAMA Ophthalmol..

[49] Di Giulio D.B., Eckburg P.B. (2004). Human monkeypox: An emerging zoonosis. Lancet Infect. Dis..

[50] Nguyen M., Doan T., Seitzman G.D. (2024). Ocular manifestations of mpox. Curr. Opin. Ophthalmol..

[51] Nguyen M.T., Mentreddy A., Schallhorn J., Chan M., Aung S., Doernberg S.B., Babik J., Miles K., Yang K., Lydon E. (2023). Isolated Ocular Mpox without Skin Lesions, United States. Emerg. Infect. Dis..

[52] Chakravarty N., Hemani D., Paravastu R., Ahmad Z., Palani S.N., Arumugaswami V., Kumar A. (2024). Mpox Virus and its ocular surface manifestations. Ocul. Surf..

[53] Ogoina D., Iroezindu M., James H.I., Oladokun R., Yinka-Ogunleye A., Wakama P., Otike-Odibi B., Usman L.M., Obazee E., Aruna O. (2020). Clinical Course and Outcome of Human Monkeypox in Nigeria. Clin. Infect. Dis..

[54] Benatti S.V., Venturelli S., Comi N., Borghi F., Paolucci S., Baldanti F. (2022). Ophthalmic manifestation of monkeypox infection. Lancet Infect. Dis..

[55] de Sousa D., Patrocínio J., Frade J., Brazão C., Mancha D., Correia C., Borges-Costa J., Filipe P. (2022). Monkeypox Diagnosis by Cutaneous and Mucosal Findings. Infect. Dis. Rep..

[56] Kaufman A.R., Chodosh J., Pineda R. (2023). Monkeypox Virus and Ophthalmology-A Primer on the 2022 Monkeypox Outbreak and Monkeypox-Related Ophthalmic Disease. JAMA Ophthalmol..

[57] Ly-Yang F., Miranda-Sánchez A., Burgos-Blasco B., Fernández-Vigo J.I., Gegúndez-Fernández J.A., Díaz-Valle D. (2022). Conjunctivitis in an Individual with Monkeypox. JAMA Ophthalmol..

[58] Janseghers L., Matamba M., Colaert J., Vandepitte J., Desmyter J. (1984). Fatal monkeypox in a child in Kikwit, Zaire. Ann. Soc. Belg. Med. Trop..

[59] Jarman E.L., Alain M., Conroy N., Omam L.A. (2022). A case report of monkeypox as a result of conflict in the context of a measles campaign. Public Health Pract..

[60] Gandhi A.P., Gupta P.C., Padhi B.K., Sandeep M., Suvvari T.K., Shamim M.A., Satapathy P., Sah R., León-Figueroa D.A., Rodriguez-Morales A.J. (2023). Ophthalmic Manifestations of the Monkeypox Virus: A Systematic Review and Meta-Analysis. Pathogens.

[61] Jezek Z., Szczeniowski M., Paluku K.M., Mutombo M. (1987). Human monkeypox: Clinical features of 282 patients. J. Infect. Dis..

[62] Domínguez García L., Gutierrez-Arroyo A., Miguel-Buckley R., Martin Ucero A., Cantizani J., Boto-de-los-Bueis A. (2024). Persistent and Severe Mpox Keratitis Despite Systemic and Topical Treatment. Cornea.

[63] Androudi S., Kaufman A.R., Kouvalakis A., Mitsios A., Sapounas S., Al-Khatib D., Schibler M., Pineda R., Baglivo E. (2024). Non-Healing Corneal Ulcer and Uveitis Following Monkeypox Disease: Diagnostic and Therapeutic Challenges. Ocul. Immunol. Inflamm..

[64] Finamor L.P.S., Mendes-Correa M.C., Rinkevicius M., Macedo G., Sabino E.C., Villas-Boas L.S., de Paula A.V., de Araujo-Heliodoro R.H., da Costa A.C., Witkin S.S. (2024). Ocular manifestations of Monkeypox virus (MPXV) infection with viral persistence in ocular samples: A case series. Int. J. Infect. Dis..

[65] Carvalho E.M., Medeiros M., Veloso V.G., Biancardi A.L., Curi A.L.L. (2024). Monkeypox Infection Causing Conjunctival Vesicles and Anterior Uveitis. Ocul. Immunol. Inflamm..

[66] Carrubba S., Geevarghese A., Solli E., Guttha S., Sims J., Sperber L., Meehan S., Ostrovsky A. (2023). Novel severe oculocutaneous manifestations of human monkeypox virus infection and their historical analogues. Lancet Infect. Dis..

[67] Hughes C., McCollum A., Pukuta E., Karhemere S., Nguete B., Shongo Lushima R., Kabamba J., Balilo M., Muyembe Tamfum J.J., Wemakoy O. (2014). Ocular complications associated with acute monkeypox virus infection, DRC. Int. J. Infect. Dis..

[68] Pazos M., Riera J., Moll-Udina A., Catala A., Narvaez S., Fuertes I., Dotti-Boada M., Petiti G., Izquierdo-Serra J., Maldonado E. (2023). Characteristics and Management of Ocular Involvement in Individuals with Monkeypox Disease. Ophthalmology.

[69] Patel A., Bilinska J., Tam J.C.H., Da Silva Fontoura D., Mason C.Y., Daunt A., Snell L.B., Murphy J., Potter J., Tuudah C. (2022). Clinical features and novel presentations of human monkeypox in a central London centre during the 2022 outbreak: Descriptive case series. BMJ.

[70] Mailhe M., Beaumont A.-L., Thy M., Le Pluart D., Perrineau S., Houhou-Fidouh N., Deconinck L., Bertin C., Ferré V.M., Cortier M. (2023). Clinical characteristics of ambulatory and hospitalized patients with monkeypox virus infection: An observational cohort study. Clin. Microbiol. Infect..

[71] Doan S., Houry R., Cristea I., Boughar B., Cochereau I., Gabison E.E., Guindolet D. (2023). Severe Corneal Involvement Associated With Mpox Infection. JAMA Ophthalmol..

[72] Rodriguez-Badillo P., Rodriguez-Aldama J.C., Gabian-Fortes L.D.C., Sifuentes-Renteria S., Valdez-Gonzalez M.T., Perez-Flores B.E., Velasco-Ramos R., Fernandez-Vizcaya O., Crabtree-Ramirez B., Perez-Barragan E. (2024). Mpox-Related Ophthalmic Disease: A Retrospective Observational Study in a Single Center in Mexico. J. Infect. Dis..

[73] Ogoina D., Dalhat M.M., Denue B.A., Okowa M., Chika-Igwenyi N.M., Yusuff H.A., Christian U.C., Adekanmbi O., Ojimba A.O., Aremu J.T. (2023). Clinical characteristics and predictors of human mpox outcome during the 2022 outbreak in Nigeria: A cohort study. Lancet Infect. Dis..

[74] Malembi E., Escrig-Sarreta R., Ntumba J., Beiras C.G., Shongo R., Bengehya J., Nselaka C., Pukuta E., Mukadi-Bamuleka D., Mulopo-Mukanya N. (2025). Clinical presentation and epidemiological assessment of confirmed human mpox cases in DR Congo: A surveillance-based observational study. Lancet.

[75] Huang C.-Y., Su S.-B., Chen K.-T. (2025). A Review of epidemiology, diagnosis, and management of Mpox: The role of One Health. Glob. Health Med..

[76] Kamoi K., Watanabe T., Uchimaru K., Okayama A., Kato S., Kawamata T., Kurozumi-Karube H., Horiguchi N., Zong Y., Yamano Y. (2022). Updates on HTLV-1 Uveitis. Viruses.

[77] Cohen-Gihon I., Israeli O., Shifman O., Erez N., Melamed S., Paran N., Beth-Din A., Zvi A. (2020). Identification and Whole-Genome Sequencing of a Monkeypox Virus Strain Isolated in Israel. Microbiol. Resour. Announc..

[78] Rayati Damavandi A., Semnani F., Hassanpour K. (2023). A Review of Monkeypox Ocular Manifestations and Complications: Insights for the 2022 Outbreak. Ophthalmol. Ther..

[79] Meduri E., Malclès A., Kecik M. (2022). Conjunctivitis with Monkeypox Virus Positive Conjunctival Swabs. Ophthalmology.

[80] Ezat A.A., Abduljalil J.M., Elghareib A.M., Samir A., Elfiky A.A. (2023). The discovery of novel antivirals for the treatment of mpox: Is drug repurposing the answer?. Expert Opin. Drug Discov..

[81] DeLaurentis C.E., Kiser J., Zucker J. (2022). New Perspectives on Antimicrobial Agents: Tecovirimat for Treatment of Human Monkeypox Virus. Antimicrob. Agents Chemother..

[82] Russo A.T., Grosenbach D.W., Honeychurch K.M., Long P.G., Hruby D.E. (2023). Overview of the regulatory approval of tecovirimat intravenous formulation for treatment of smallpox: Potential impact on smallpox outbreak response capabilities, and future tecovirimat development potential. Expert Rev. Anti-Infect. Infect. Ther..

[83] Shabil M., Khatib M.N., Ballal S., Bansal P., Tomar B.S., Ashraf A., Kumar M.R., Sinha A., Rawat P., Gaidhane A.M. (2024). Effectiveness of Tecovirimat in Mpox Cases: A Systematic Review of Current Evidence. J. Med. Virol..

[84] Milligan A.L., Koay S.Y., Dunning J. (2022). Monkeypox as an emerging infectious disease: The ophthalmic implications. Br. J. Ophthalmol..

[85] Rizk J.G., Lippi G., Henry B.M., Forthal D.N., Rizk Y. (2022). Prevention and Treatment of Monkeypox. Drugs.

[86] Trawally Flores A., Guedes Guedes I.I., Espinoza González J.P., Jerez Olivera E., Siguero Martín L., Pérez Álvarez J. (2024). Ocular involvement secondary to Monkeypox virus infection. Arch. Soc. Española Oftalmol..

[87] Pischel L., Martini B.A., Yu N., Cacesse D., Tracy M., Kharbanda K., Ahmed N., Patel K.M., Grimshaw A.A., Malik A.A. (2024). Vaccine effectiveness of 3rd generation mpox vaccines against mpox and disease severity: A systematic review and meta-analysis. Vaccine.

[88] Rao A.K., Petersen B.W., Whitehill F., Razeq J.H., Isaacs S.N., Merchlinsky M.J., Campos-Outcalt D., Morgan R.L., Damon I., Sanchez P.J. (2022). Use of JYNNEOS (Smallpox and Monkeypox Vaccine, Live, Nonreplicating) for Preexposure Vaccination of Persons at Risk for Occupational Exposure to Orthopoxviruses: Recommendations of the Advisory Committee on Immunization Practices—United States, 2022. MMWR Morb. Mortal. Wkly. Rep..

[89] Wang X., Gu Z., Sheng S., Song R., Jin R. (2024). The Current State and Progress of Mpox Vaccine Research. China CDC Wkly..

[90] Neff J.M., Lane J.M., Pert J.H., Moore R., Millar J.D., Henderson D.A. (1967). Complications of smallpox vaccination: National survey in the United States, 1963. N. Engl. J. Med..

[91] Pepose J.S., Margolis T.P., LaRussa P., Pavan-Langston D. (2003). Ocular complications of smallpox vaccination. Am. J. Ophthalmol..

[92] Cono J., Casey C.G., Bell D.M., Centers for Disease Control and Prevention (2003). Smallpox vaccination and adverse reactions. MMWR Recomm. Rep..

[93] Malik S., Ahmad T., Ahsan O., Muhammad K., Waheed Y. (2023). Recent Developments in Mpox Prevention and Treatment Options. Vaccines.

[94] Gupta P.C., Sharma N., Rai S., Mishra P., Bachheti R.K., Bachheti A., Husen A. (2024). Use of Smart Silver Nanoparticles in Drug Delivery System. Metal and Metal-Oxide Based Nanomaterials: Synthesis, Agricultural, Biomedical and Environmental Interventions.

[95] Verma A., Nazli Khatib M., Datt Sharma G., Pratap Singh M., Bushi G., Ballal S., Kumar S., Bhat M., Sharma S., Ndabashinze R. (2024). Mpox 2024: New variant, new challenges, and the looming pandemic. Clin. Infect. Pract..

